# Corrigendum: Sparse testing designs for optimizing predictive ability in sugarcane populations

**DOI:** 10.3389/fpls.2024.1520147

**Published:** 2024-11-15

**Authors:** Julian Garcia-Abadillo, Paul Adunola, Fernando Silva Aguilar, Jhon Henry Trujillo-Montenegro, John Jaime Riascos, Reyna Persa, Julio Isidro y Sanchez, Diego Jarquín

**Affiliations:** ^1^ Centro de Biotecnología y Genómica de Plantas, Universidad Politécnica de Madrid, Madrid, Spain; ^2^ Agronomy Department, University of Florida, Gainesville, FL, United States; ^3^ Horticultural Sciences Department, University of Florida, Gainesville, FL, United States; ^4^ Colombian Sugarcane Research Center, Cenicaña, Cali, Valle del Cauca, Colombia

**Keywords:** genomic selection GS, genomic prediction GP, sparse testing designs, optimization, sugarcane breeding

In the published article, there was an error in the **Funding** statement regarding the non-inclusion of a funding agency. The original text was “The author(s) declare financial support was received for the research, authorship, and/or publication of this article. JG-A was funded a UPM predoctoral grant as part of the program “Programa Propio I +D+i” financed by the Universidad Politécnica de Madrid. JIS was supported by the Beatriz Galindo Program (BEAGAL18/00115) from the Ministerio de Educación y Formación Profesional of Spain and the Severo Ochoa Program for Centers of Excellence in R&D from the “Agencia Estatal de Investigación” of Spain, grant SEV-2016SEV- -0672 (2017SEV- -2021)) to the CBGP. JIS was also supported by Grant PID2021-123718OB-I00 funded by MCIN/AEI/10.13039/501100011033 and by “ERDF A way of making Europe, CEX2020-000999-S”.”” The correct Funding statement appears below.

“The author(s) declared financial support was received for the research, authorship, and/or publication of this article. The generation and collection of the data used in this work was supported with funding from Cenicaña provided by the sugarcane mills and producers from the Cauca River valley in Colombia. JG-A was funded a UPM predoctoral grant as part of the program “Programa Propio I +D+i” financed by the Universidad Politécnica de Madrid. JIS was supported by the Beatriz Galindo Program (BEAGAL18/00115) from the Ministerio de Educación y Formación Profesional of Spain and the Severo Ochoa Program for Centers of Excellence in R&D from the “Agencia Estatal de Investigación” of Spain, grant SEV-2016SEV- -0672 (2017SEV- -2021)) to the CBGP. JIS was also supported by Grant PID2021-123718OB-I00 funded by MCIN/AEI/10.13039/501100011033 and by “ERDF A way of making Europe, CEX2020-000999-S”.””

In the published article, there was an error caused by not including the **Acknowledgments** section.

A correction has been made to **Acknowledgments**.

The corrected sentence appears below:

“The authors would like to thank the mills and growers who have supported Cenicaña for more than 46 years. Additionally, we would like to give special thanks to the plant breeders at Cenicaña who have been actively releasing commercial varieties in Colombia.”

The authors apologize for these errors and state that this does not change the scientific conclusions of the article in any way. The original article has been updated.

