# Supplementary material for: Sparse testing designs for optimizing predictive ability in sugarcane populations
Source: Front Plant Sci. 2024 Jul 23;15:1400000. doi: 10.3389/fpls.2024.1400000 (PMC11300217; doi:10.3389/fpls.2024.1400000)
Supplement: Supplementary file 1 [file Table1.docx]

Supplementary Material

Supplementary Table 1. Average Pearson’s correlation values between observed and predicted phenotypic values for 10 replicates for different sparse testing designs and environments (E1: Balsora 16, E2: Balsora 17, E3: Taula 18, E4: Taula 19, E5: Porvenir 20, E6: Porvenir 21).

| Model | Trait | SS | Design | E1 | E2 | E3 | E4 | E5 | E6 | Mean |
| --- | --- | --- | --- | --- | --- | --- | --- | --- | --- | --- |
| M1: E + L | SA | 31 | 31/0 | 0.65 | 0.62 | 0.52 | 0.5 | 0.43 | 0.61 | 0.56 |
| M1: E + L | SA | 31 | 28/3 | 0.59 | 0.59 | 0.51 | 0.48 | 0.46 | 0.6 | 0.54 |
| M1: E + L | SA | 31 | 24/7 | 0.57 | 0.52 | 0.49 | 0.45 | 0.41 | 0.54 | 0.5 |
| M1: E + L | SA | 31 | 20/11 | 0.51 | 0.48 | 0.42 | 0.4 | 0.4 | 0.49 | 0.45 |
| M1: E + L | SA | 31 | 16/15 | 0.43 | 0.39 | 0.36 | 0.32 | 0.25 | 0.42 | 0.36 |
| M1: E + L | SA | 31 | 12/19 | 0.39 | 0.4 | 0.37 | 0.32 | 0.23 | 0.4 | 0.35 |
| M1: E + L | SA | 31 | 8/23 | 0.29 | 0.26 | 0.25 | 0.21 | 0.25 | 0.25 | 0.25 |
| M1: E + L | SA | 31 | 4/27 | 0.16 | 0.15 | 0.13 | 0.11 | 0.12 | 0.13 | 0.13 |
| M1: E + L | SA | 31 | 0/31 | 0 | 0 | 0.02 | 0.01 | 0.03 | 0 | 0.01 |
| M1: E + L | TCH | 31 | 31/0 | 0.36 | 0.37 | 0.2 | 0.3 | 0.3 | 0.38 | 0.32 |
| M1: E + L | TCH | 31 | 28/3 | 0.32 | 0.35 | 0.21 | 0.31 | 0.29 | 0.39 | 0.31 |
| M1: E + L | TCH | 31 | 24/7 | 0.29 | 0.34 | 0.21 | 0.3 | 0.27 | 0.33 | 0.29 |
| M1: E + L | TCH | 31 | 20/11 | 0.26 | 0.27 | 0.19 | 0.32 | 0.24 | 0.32 | 0.27 |
| M1: E + L | TCH | 31 | 16/15 | 0.23 | 0.28 | 0.13 | 0.26 | 0.17 | 0.27 | 0.22 |
| M1: E + L | TCH | 31 | 12/19 | 0.23 | 0.22 | 0.14 | 0.18 | 0.18 | 0.25 | 0.2 |
| M1: E + L | TCH | 31 | 8/23 | 0.22 | 0.2 | 0.1 | 0.15 | 0.14 | 0.21 | 0.17 |
| M1: E + L | TCH | 31 | 4/27 | 0.08 | 0.11 | 0.06 | 0.08 | 0.08 | 0.13 | 0.09 |
| M1: E + L | TCH | 31 | 0/31 | 0.02 | 0.03 | 0.04 | 0.03 | 0.03 | 0.05 | 0.03 |
| M2: E + L + g | SA | 31 | 31/0 | 0.69 | 0.66 | 0.55 | 0.51 | 0.45 | 0.63 | 0.58 |
| M2: E + L + g | SA | 31 | 28/3 | 0.69 | 0.67 | 0.56 | 0.52 | 0.48 | 0.67 | 0.6 |
| M2: E + L + g | SA | 31 | 24/7 | 0.7 | 0.64 | 0.56 | 0.51 | 0.45 | 0.65 | 0.59 |
| M2: E + L + g | SA | 31 | 20/11 | 0.67 | 0.62 | 0.51 | 0.47 | 0.46 | 0.61 | 0.56 |
| M2: E + L + g | SA | 31 | 16/15 | 0.6 | 0.56 | 0.47 | 0.41 | 0.34 | 0.56 | 0.49 |
| M2: E + L + g | SA | 31 | 12/19 | 0.63 | 0.6 | 0.52 | 0.43 | 0.33 | 0.58 | 0.52 |
| M2: E + L + g | SA | 31 | 8/23 | 0.53 | 0.48 | 0.41 | 0.32 | 0.36 | 0.45 | 0.42 |
| M2: E + L + g | SA | 31 | 4/27 | 0.46 | 0.42 | 0.33 | 0.25 | 0.26 | 0.38 | 0.35 |
| M2: E + L + g | SA | 31 | 0/31 | 0.34 | 0.31 | 0.26 | 0.17 | 0.26 | 0.27 | 0.27 |
| M2: E + L + g | TCH | 31 | 31/0 | 0.37 | 0.38 | 0.21 | 0.31 | 0.31 | 0.39 | 0.33 |
| M2: E + L + g | TCH | 31 | 28/3 | 0.33 | 0.37 | 0.22 | 0.32 | 0.31 | 0.4 | 0.33 |
| M2: E + L + g | TCH | 31 | 24/7 | 0.31 | 0.37 | 0.23 | 0.32 | 0.28 | 0.36 | 0.31 |
| M2: E + L + g | TCH | 31 | 20/11 | 0.28 | 0.3 | 0.21 | 0.33 | 0.26 | 0.34 | 0.29 |
| M2: E + L + g | TCH | 31 | 16/15 | 0.25 | 0.31 | 0.15 | 0.28 | 0.19 | 0.29 | 0.24 |
| M2: E + L + g | TCH | 31 | 12/19 | 0.26 | 0.26 | 0.17 | 0.2 | 0.19 | 0.28 | 0.23 |
| M2: E + L + g | TCH | 31 | 8/23 | 0.25 | 0.24 | 0.12 | 0.17 | 0.17 | 0.24 | 0.2 |
| M2: E + L + g | TCH | 31 | 4/27 | 0.11 | 0.16 | 0.08 | 0.1 | 0.1 | 0.16 | 0.12 |
| M2: E + L + g | TCH | 31 | 0/31 | 0.1 | 0.1 | 0.02 | 0.03 | 0.09 | 0.1 | 0.07 |
| M3: E + L + g + gE | SA | 31 | 31/0 | 0.73 | 0.69 | 0.56 | 0.52 | 0.46 | 0.66 | 0.6 |
| M3: E + L + g + gE | SA | 31 | 28/3 | 0.73 | 0.7 | 0.57 | 0.51 | 0.48 | 0.68 | 0.61 |
| M3: E + L + g + gE | SA | 31 | 24/7 | 0.73 | 0.68 | 0.56 | 0.51 | 0.46 | 0.66 | 0.6 |
| M3: E + L + g + gE | SA | 31 | 20/11 | 0.71 | 0.64 | 0.51 | 0.46 | 0.44 | 0.62 | 0.56 |
| M3: E + L + g + gE | SA | 31 | 16/15 | 0.64 | 0.59 | 0.45 | 0.41 | 0.33 | 0.54 | 0.49 |
| M3: E + L + g + gE | SA | 31 | 12/19 | 0.69 | 0.61 | 0.5 | 0.41 | 0.33 | 0.6 | 0.52 |
| M3: E + L + g + gE | SA | 31 | 8/23 | 0.62 | 0.54 | 0.36 | 0.26 | 0.35 | 0.47 | 0.43 |
| M3: E + L + g + gE | SA | 31 | 4/27 | 0.53 | 0.45 | 0.21 | 0.18 | 0.23 | 0.38 | 0.33 |
| M3: E + L + g + gE | SA | 31 | 0/31 | 0.42 | 0.37 | 0.04 | 0.03 | 0.14 | 0.18 | 0.2 |
| M3: E + L + g + gE | TCH | 31 | 31/0 | 0.37 | 0.42 | 0.21 | 0.31 | 0.34 | 0.4 | 0.34 |
| M3: E + L + g + gE | TCH | 31 | 28/3 | 0.33 | 0.4 | 0.22 | 0.31 | 0.32 | 0.41 | 0.33 |
| M3: E + L + g + gE | TCH | 31 | 24/7 | 0.31 | 0.4 | 0.23 | 0.32 | 0.3 | 0.36 | 0.32 |
| M3: E + L + g + gE | TCH | 31 | 20/11 | 0.27 | 0.35 | 0.2 | 0.32 | 0.29 | 0.35 | 0.3 |
| M3: E + L + g + gE | TCH | 31 | 16/15 | 0.24 | 0.34 | 0.14 | 0.27 | 0.22 | 0.29 | 0.25 |
| M3: E + L + g + gE | TCH | 31 | 12/19 | 0.24 | 0.29 | 0.16 | 0.19 | 0.22 | 0.28 | 0.23 |
| M3: E + L + g + gE | TCH | 31 | 8/23 | 0.19 | 0.32 | 0.09 | 0.15 | 0.21 | 0.27 | 0.2 |
| M3: E + L + g + gE | TCH | 31 | 4/27 | 0.1 | 0.25 | 0.05 | 0.09 | 0.16 | 0.13 | 0.13 |
| M3: E + L + g + gE | TCH | 31 | 0/31 | 0.1 | 0.15 | 0.06 | -0.01 | 0.11 | 0.01 | 0.07 |
| M1: E + L | SA | 28 | 28/0 | 0.62 | 0.55 | 0.51 | 0.42 | 0.41 | 0.59 | 0.52 |
| M1: E + L | SA | 28 | 24/4 | 0.54 | 0.53 | 0.45 | 0.45 | 0.41 | 0.55 | 0.49 |
| M1: E + L | SA | 28 | 20/8 | 0.54 | 0.48 | 0.44 | 0.43 | 0.38 | 0.49 | 0.46 |
| M1: E + L | SA | 28 | 16/12 | 0.47 | 0.44 | 0.36 | 0.37 | 0.36 | 0.44 | 0.41 |
| M1: E + L | SA | 28 | 12/16 | 0.36 | 0.32 | 0.29 | 0.28 | 0.22 | 0.34 | 0.3 |
| M1: E + L | SA | 28 | 8/20 | 0.37 | 0.34 | 0.33 | 0.3 | 0.2 | 0.35 | 0.32 |
| M1: E + L | SA | 28 | 4/24 | 0.21 | 0.18 | 0.19 | 0.16 | 0.2 | 0.18 | 0.19 |
| M1: E + L | SA | 28 | 0/28 | -0.02 | -0.02 | 0.03 | 0.04 | 0.02 | -0.01 | 0.01 |
| M1: E + L | TCH | 28 | 28/0 | 0.33 | 0.34 | 0.2 | 0.32 | 0.28 | 0.36 | 0.3 |
| M1: E + L | TCH | 28 | 24/4 | 0.28 | 0.33 | 0.2 | 0.3 | 0.28 | 0.35 | 0.29 |
| M1: E + L | TCH | 28 | 20/8 | 0.28 | 0.32 | 0.21 | 0.29 | 0.25 | 0.32 | 0.28 |
| M1: E + L | TCH | 28 | 16/12 | 0.24 | 0.25 | 0.16 | 0.27 | 0.22 | 0.28 | 0.24 |
| M1: E + L | TCH | 28 | 12/16 | 0.22 | 0.23 | 0.11 | 0.23 | 0.15 | 0.22 | 0.19 |
| M1: E + L | TCH | 28 | 8/20 | 0.19 | 0.2 | 0.1 | 0.16 | 0.14 | 0.2 | 0.16 |
| M1: E + L | TCH | 28 | 4/24 | 0.15 | 0.13 | 0.1 | 0.11 | 0.1 | 0.16 | 0.13 |
| M1: E + L | TCH | 28 | 0/28 | 0.04 | 0.05 | 0.02 | 0 | -0.02 | 0.01 | 0.02 |
| M2: E + L + g | SA | 28 | 28/0 | 0.68 | 0.61 | 0.54 | 0.46 | 0.44 | 0.64 | 0.56 |
| M2: E + L + g | SA | 28 | 24/4 | 0.66 | 0.64 | 0.53 | 0.5 | 0.46 | 0.65 | 0.57 |
| M2: E + L + g | SA | 28 | 20/8 | 0.68 | 0.62 | 0.53 | 0.5 | 0.44 | 0.61 | 0.56 |
| M2: E + L + g | SA | 28 | 16/12 | 0.65 | 0.6 | 0.47 | 0.45 | 0.42 | 0.59 | 0.53 |
| M2: E + L + g | SA | 28 | 12/16 | 0.54 | 0.5 | 0.42 | 0.37 | 0.31 | 0.48 | 0.44 |
| M2: E + L + g | SA | 28 | 8/20 | 0.6 | 0.56 | 0.47 | 0.4 | 0.32 | 0.54 | 0.48 |
| M2: E + L + g | SA | 28 | 4/24 | 0.46 | 0.4 | 0.35 | 0.27 | 0.33 | 0.39 | 0.37 |
| M2: E + L + g | SA | 28 | 0/28 | 0.42 | 0.4 | 0.31 | 0.24 | 0.24 | 0.38 | 0.33 |
| M2: E + L + g | TCH | 28 | 28/0 | 0.34 | 0.35 | 0.21 | 0.32 | 0.29 | 0.37 | 0.31 |
| M2: E + L + g | TCH | 28 | 24/4 | 0.3 | 0.35 | 0.21 | 0.31 | 0.3 | 0.37 | 0.31 |
| M2: E + L + g | TCH | 28 | 20/8 | 0.3 | 0.35 | 0.23 | 0.3 | 0.27 | 0.35 | 0.3 |
| M2: E + L + g | TCH | 28 | 16/12 | 0.26 | 0.28 | 0.19 | 0.29 | 0.23 | 0.31 | 0.26 |
| M2: E + L + g | TCH | 28 | 12/16 | 0.23 | 0.27 | 0.14 | 0.25 | 0.16 | 0.25 | 0.22 |
| M2: E + L + g | TCH | 28 | 8/20 | 0.22 | 0.24 | 0.12 | 0.18 | 0.16 | 0.23 | 0.19 |
| M2: E + L + g | TCH | 28 | 4/24 | 0.17 | 0.14 | 0.12 | 0.12 | 0.13 | 0.19 | 0.15 |
| M2: E + L + g | TCH | 28 | 0/28 | 0.08 | 0.06 | 0.05 | 0 | 0.13 | 0.09 | 0.07 |
| M3: E + L + g + gE | SA | 28 | 28/0 | 0.72 | 0.67 | 0.56 | 0.47 | 0.44 | 0.67 | 0.59 |
| M3: E + L + g + gE | SA | 28 | 24/4 | 0.7 | 0.67 | 0.53 | 0.5 | 0.46 | 0.66 | 0.59 |
| M3: E + L + g + gE | SA | 28 | 20/8 | 0.69 | 0.64 | 0.54 | 0.5 | 0.43 | 0.62 | 0.57 |
| M3: E + L + g + gE | SA | 28 | 16/12 | 0.7 | 0.63 | 0.47 | 0.45 | 0.41 | 0.61 | 0.55 |
| M3: E + L + g + gE | SA | 28 | 12/16 | 0.57 | 0.53 | 0.4 | 0.34 | 0.32 | 0.47 | 0.44 |
| M3: E + L + g + gE | SA | 28 | 8/20 | 0.66 | 0.59 | 0.45 | 0.38 | 0.31 | 0.53 | 0.49 |
| M3: E + L + g + gE | SA | 28 | 4/24 | 0.53 | 0.47 | 0.31 | 0.22 | 0.24 | 0.41 | 0.36 |
| M3: E + L + g + gE | SA | 28 | 0/28 | 0.45 | 0.4 | 0.12 | 0.04 | 0.14 | 0.23 | 0.23 |
| M3: E + L + g + gE | TCH | 28 | 28/0 | 0.34 | 0.4 | 0.21 | 0.32 | 0.31 | 0.38 | 0.33 |
| M3: E + L + g + gE | TCH | 28 | 24/4 | 0.29 | 0.38 | 0.21 | 0.31 | 0.31 | 0.39 | 0.31 |
| M3: E + L + g + gE | TCH | 28 | 20/8 | 0.3 | 0.39 | 0.23 | 0.3 | 0.28 | 0.34 | 0.31 |
| M3: E + L + g + gE | TCH | 28 | 16/12 | 0.26 | 0.33 | 0.18 | 0.28 | 0.26 | 0.32 | 0.27 |
| M3: E + L + g + gE | TCH | 28 | 12/16 | 0.23 | 0.3 | 0.14 | 0.24 | 0.18 | 0.25 | 0.22 |
| M3: E + L + g + gE | TCH | 28 | 8/20 | 0.21 | 0.29 | 0.12 | 0.17 | 0.19 | 0.23 | 0.2 |
| M3: E + L + g + gE | TCH | 28 | 4/24 | 0.12 | 0.21 | 0.12 | 0.11 | 0.16 | 0.18 | 0.15 |
| M3: E + L + g + gE | TCH | 28 | 0/28 | 0.08 | 0.19 | 0.02 | 0.04 | 0.09 | 0.09 | 0.08 |
| M1: E + L | SA | 24 | 24/0 | 0.56 | 0.56 | 0.47 | 0.43 | 0.39 | 0.53 | 0.49 |
| M1: E + L | SA | 24 | 20/4 | 0.49 | 0.47 | 0.4 | 0.41 | 0.37 | 0.5 | 0.44 |
| M1: E + L | SA | 24 | 16/8 | 0.45 | 0.4 | 0.36 | 0.35 | 0.31 | 0.41 | 0.38 |
| M1: E + L | SA | 24 | 12/12 | 0.42 | 0.4 | 0.34 | 0.32 | 0.31 | 0.4 | 0.37 |
| M1: E + L | SA | 24 | 8/16 | 0.28 | 0.21 | 0.24 | 0.23 | 0.19 | 0.26 | 0.24 |
| M1: E + L | SA | 24 | 4/20 | 0.2 | 0.23 | 0.22 | 0.21 | 0.1 | 0.22 | 0.2 |
| M1: E + L | SA | 24 | 0/24 | -0.01 | -0.01 | -0.05 | 0.01 | -0.01 | -0.01 | -0.01 |
| M1: E + L | TCH | 24 | 24/0 | 0.28 | 0.31 | 0.17 | 0.27 | 0.28 | 0.35 | 0.28 |
| M1: E + L | TCH | 24 | 20/4 | 0.25 | 0.29 | 0.19 | 0.26 | 0.25 | 0.32 | 0.26 |
| M1: E + L | TCH | 24 | 16/8 | 0.26 | 0.27 | 0.18 | 0.23 | 0.22 | 0.3 | 0.24 |
| M1: E + L | TCH | 24 | 12/12 | 0.21 | 0.21 | 0.13 | 0.22 | 0.18 | 0.23 | 0.2 |
| M1: E + L | TCH | 24 | 8/16 | 0.15 | 0.19 | 0.09 | 0.19 | 0.12 | 0.19 | 0.15 |
| M1: E + L | TCH | 24 | 4/20 | 0.13 | 0.1 | 0.08 | 0.13 | 0.08 | 0.11 | 0.1 |
| M1: E + L | TCH | 24 | 0/24 | -0.04 | -0.03 | -0.01 | 0 | 0.01 | 0 | -0.01 |
| M2: E + L + g | SA | 24 | 24/0 | 0.67 | 0.65 | 0.54 | 0.48 | 0.44 | 0.61 | 0.57 |
| M2: E + L + g | SA | 24 | 20/4 | 0.64 | 0.6 | 0.49 | 0.47 | 0.43 | 0.61 | 0.54 |
| M2: E + L + g | SA | 24 | 16/8 | 0.63 | 0.57 | 0.47 | 0.43 | 0.39 | 0.56 | 0.51 |
| M2: E + L + g | SA | 24 | 12/12 | 0.62 | 0.57 | 0.46 | 0.41 | 0.39 | 0.56 | 0.5 |
| M2: E + L + g | SA | 24 | 8/16 | 0.47 | 0.42 | 0.37 | 0.32 | 0.29 | 0.42 | 0.38 |
| M2: E + L + g | SA | 24 | 4/20 | 0.44 | 0.42 | 0.36 | 0.3 | 0.24 | 0.4 | 0.36 |
| M2: E + L + g | SA | 24 | 0/24 | 0.41 | 0.36 | 0.29 | 0.2 | 0.27 | 0.34 | 0.31 |
| M2: E + L + g | TCH | 24 | 24/0 | 0.3 | 0.33 | 0.18 | 0.27 | 0.29 | 0.37 | 0.29 |
| M2: E + L + g | TCH | 24 | 20/4 | 0.27 | 0.32 | 0.21 | 0.28 | 0.27 | 0.34 | 0.28 |
| M2: E + L + g | TCH | 24 | 16/8 | 0.28 | 0.3 | 0.2 | 0.25 | 0.23 | 0.31 | 0.26 |
| M2: E + L + g | TCH | 24 | 12/12 | 0.23 | 0.24 | 0.14 | 0.23 | 0.19 | 0.25 | 0.21 |
| M2: E + L + g | TCH | 24 | 8/16 | 0.18 | 0.24 | 0.12 | 0.22 | 0.13 | 0.22 | 0.18 |
| M2: E + L + g | TCH | 24 | 4/20 | 0.18 | 0.17 | 0.1 | 0.17 | 0.1 | 0.16 | 0.15 |
| M2: E + L + g | TCH | 24 | 0/24 | 0.07 | 0.11 | 0.06 | 0.06 | 0.06 | 0.09 | 0.07 |
| M3: E + L + g + gE | SA | 24 | 24/0 | 0.72 | 0.68 | 0.56 | 0.49 | 0.45 | 0.65 | 0.59 |
| M3: E + L + g + gE | SA | 24 | 20/4 | 0.66 | 0.63 | 0.5 | 0.46 | 0.44 | 0.63 | 0.55 |
| M3: E + L + g + gE | SA | 24 | 16/8 | 0.65 | 0.6 | 0.47 | 0.42 | 0.38 | 0.55 | 0.51 |
| M3: E + L + g + gE | SA | 24 | 12/12 | 0.62 | 0.56 | 0.44 | 0.4 | 0.37 | 0.56 | 0.49 |
| M3: E + L + g + gE | SA | 24 | 8/16 | 0.52 | 0.47 | 0.31 | 0.28 | 0.28 | 0.4 | 0.38 |
| M3: E + L + g + gE | SA | 24 | 4/20 | 0.5 | 0.47 | 0.3 | 0.25 | 0.2 | 0.4 | 0.35 |
| M3: E + L + g + gE | SA | 24 | 0/24 | 0.45 | 0.42 | 0.15 | 0.05 | 0.04 | 0.2 | 0.22 |
| M3: E + L + g + gE | TCH | 24 | 24/0 | 0.31 | 0.35 | 0.19 | 0.27 | 0.31 | 0.38 | 0.3 |
| M3: E + L + g + gE | TCH | 24 | 20/4 | 0.27 | 0.37 | 0.2 | 0.26 | 0.28 | 0.36 | 0.29 |
| M3: E + L + g + gE | TCH | 24 | 16/8 | 0.26 | 0.32 | 0.18 | 0.24 | 0.26 | 0.32 | 0.26 |
| M3: E + L + g + gE | TCH | 24 | 12/12 | 0.2 | 0.27 | 0.14 | 0.23 | 0.22 | 0.26 | 0.22 |
| M3: E + L + g + gE | TCH | 24 | 8/16 | 0.13 | 0.29 | 0.11 | 0.19 | 0.16 | 0.21 | 0.18 |
| M3: E + L + g + gE | TCH | 24 | 4/20 | 0.15 | 0.24 | 0.09 | 0.13 | 0.14 | 0.16 | 0.15 |
| M3: E + L + g + gE | TCH | 24 | 0/24 | 0.03 | 0.18 | 0.06 | -0.02 | 0.1 | 0.08 | 0.07 |
| M1: E + L | SA | 20 | 20/0 | 0.45 | 0.49 | 0.43 | 0.38 | 0.34 | 0.49 | 0.43 |
| M1: E + L | SA | 20 | 16/4 | 0.42 | 0.4 | 0.35 | 0.35 | 0.35 | 0.45 | 0.39 |
| M1: E + L | SA | 20 | 12/8 | 0.39 | 0.33 | 0.31 | 0.31 | 0.26 | 0.36 | 0.33 |
| M1: E + L | SA | 20 | 8/12 | 0.35 | 0.35 | 0.3 | 0.3 | 0.26 | 0.36 | 0.32 |
| M1: E + L | SA | 20 | 4/16 | 0.24 | 0.16 | 0.2 | 0.19 | 0.14 | 0.23 | 0.19 |
| M1: E + L | SA | 20 | 0/20 | -0.03 | -0.01 | -0.02 | 0.01 | -0.02 | -0.02 | -0.01 |
| M1: E + L | TCH | 20 | 20/0 | 0.26 | 0.29 | 0.14 | 0.27 | 0.27 | 0.32 | 0.26 |
| M1: E + L | TCH | 20 | 16/4 | 0.21 | 0.26 | 0.18 | 0.24 | 0.23 | 0.27 | 0.23 |
| M1: E + L | TCH | 20 | 12/8 | 0.19 | 0.25 | 0.17 | 0.21 | 0.18 | 0.25 | 0.21 |
| M1: E + L | TCH | 20 | 8/12 | 0.18 | 0.18 | 0.11 | 0.18 | 0.14 | 0.19 | 0.16 |
| M1: E + L | TCH | 20 | 4/16 | 0.11 | 0.12 | 0.08 | 0.11 | 0.07 | 0.14 | 0.1 |
| M1: E + L | TCH | 20 | 0/20 | -0.01 | 0.03 | -0.02 | 0 | 0.02 | 0.04 | 0.01 |
| M2: E + L + g | SA | 20 | 20/0 | 0.59 | 0.59 | 0.5 | 0.44 | 0.39 | 0.59 | 0.52 |
| M2: E + L + g | SA | 20 | 16/4 | 0.58 | 0.55 | 0.45 | 0.42 | 0.42 | 0.57 | 0.5 |
| M2: E + L + g | SA | 20 | 12/8 | 0.56 | 0.5 | 0.42 | 0.39 | 0.34 | 0.5 | 0.45 |
| M2: E + L + g | SA | 20 | 8/12 | 0.58 | 0.54 | 0.44 | 0.4 | 0.35 | 0.54 | 0.47 |
| M2: E + L + g | SA | 20 | 4/16 | 0.41 | 0.35 | 0.31 | 0.26 | 0.23 | 0.36 | 0.32 |
| M2: E + L + g | SA | 20 | 0/20 | 0.25 | 0.23 | 0.21 | 0.13 | 0.18 | 0.22 | 0.2 |
| M2: E + L + g | TCH | 20 | 20/0 | 0.28 | 0.31 | 0.15 | 0.28 | 0.28 | 0.33 | 0.27 |
| M2: E + L + g | TCH | 20 | 16/4 | 0.24 | 0.28 | 0.19 | 0.25 | 0.25 | 0.29 | 0.25 |
| M2: E + L + g | TCH | 20 | 12/8 | 0.22 | 0.27 | 0.18 | 0.23 | 0.2 | 0.27 | 0.23 |
| M2: E + L + g | TCH | 20 | 8/12 | 0.2 | 0.22 | 0.14 | 0.2 | 0.15 | 0.22 | 0.19 |
| M2: E + L + g | TCH | 20 | 4/16 | 0.15 | 0.16 | 0.1 | 0.13 | 0.08 | 0.16 | 0.13 |
| M2: E + L + g | TCH | 20 | 0/20 | 0.03 | 0.06 | 0.03 | 0.05 | 0.08 | 0.07 | 0.05 |
| M3: E + L + g + gE | SA | 20 | 20/0 | 0.68 | 0.59 | 0.52 | 0.43 | 0.39 | 0.6 | 0.54 |
| M3: E + L + g + gE | SA | 20 | 16/4 | 0.6 | 0.59 | 0.43 | 0.41 | 0.41 | 0.57 | 0.5 |
| M3: E + L + g + gE | SA | 20 | 12/8 | 0.61 | 0.52 | 0.41 | 0.34 | 0.31 | 0.48 | 0.44 |
| M3: E + L + g + gE | SA | 20 | 8/12 | 0.64 | 0.57 | 0.4 | 0.37 | 0.34 | 0.56 | 0.48 |
| M3: E + L + g + gE | SA | 20 | 4/16 | 0.45 | 0.42 | 0.25 | 0.22 | 0.22 | 0.35 | 0.32 |
| M3: E + L + g + gE | SA | 20 | 0/20 | 0.33 | 0.26 | -0.06 | -0.03 | 0.13 | 0.11 | 0.13 |
| M3: E + L + g + gE | TCH | 20 | 20/0 | 0.28 | 0.33 | 0.15 | 0.28 | 0.3 | 0.34 | 0.28 |
| M3: E + L + g + gE | TCH | 20 | 16/4 | 0.22 | 0.33 | 0.19 | 0.25 | 0.26 | 0.31 | 0.26 |
| M3: E + L + g + gE | TCH | 20 | 12/8 | 0.19 | 0.3 | 0.19 | 0.23 | 0.22 | 0.26 | 0.23 |
| M3: E + L + g + gE | TCH | 20 | 8/12 | 0.19 | 0.26 | 0.12 | 0.19 | 0.18 | 0.23 | 0.19 |
| M3: E + L + g + gE | TCH | 20 | 4/16 | 0.11 | 0.22 | 0.07 | 0.1 | 0.13 | 0.15 | 0.13 |
| M3: E + L + g + gE | TCH | 20 | 0/20 | 0.04 | 0.13 | 0.05 | 0.02 | 0.1 | 0.04 | 0.06 |
| M1: E + L | SA | 16 | 16/0 | 0.41 | 0.41 | 0.36 | 0.31 | 0.32 | 0.36 | 0.36 |
| M1: E + L | SA | 16 | 12/4 | 0.34 | 0.31 | 0.31 | 0.3 | 0.29 | 0.38 | 0.32 |
| M1: E + L | SA | 16 | 8/8 | 0.29 | 0.27 | 0.24 | 0.24 | 0.21 | 0.27 | 0.25 |
| M1: E + L | SA | 16 | 4/12 | 0.19 | 0.2 | 0.17 | 0.18 | 0.19 | 0.25 | 0.2 |
| M1: E + L | SA | 16 | 0/16 | -0.01 | -0.02 | -0.03 | -0.04 | -0.01 | -0.02 | -0.02 |
| M1: E + L | TCH | 16 | 16/0 | 0.22 | 0.23 | 0.11 | 0.2 | 0.17 | 0.23 | 0.19 |
| M1: E + L | TCH | 16 | 12/4 | 0.18 | 0.2 | 0.16 | 0.22 | 0.18 | 0.24 | 0.2 |
| M1: E + L | TCH | 16 | 8/8 | 0.17 | 0.21 | 0.12 | 0.15 | 0.16 | 0.2 | 0.17 |
| M1: E + L | TCH | 16 | 4/12 | 0.11 | 0.1 | 0.05 | 0.09 | 0.08 | 0.13 | 0.09 |
| M1: E + L | TCH | 16 | 0/16 | -0.07 | -0.05 | -0.02 | -0.02 | -0.01 | -0.03 | -0.03 |
| M2: E + L + g | SA | 16 | 16/0 | 0.55 | 0.52 | 0.44 | 0.39 | 0.37 | 0.47 | 0.46 |
| M2: E + L + g | SA | 16 | 12/4 | 0.51 | 0.47 | 0.4 | 0.37 | 0.36 | 0.5 | 0.44 |
| M2: E + L + g | SA | 16 | 8/8 | 0.48 | 0.45 | 0.36 | 0.33 | 0.3 | 0.43 | 0.39 |
| M2: E + L + g | SA | 16 | 4/12 | 0.37 | 0.34 | 0.28 | 0.26 | 0.27 | 0.38 | 0.32 |
| M2: E + L + g | SA | 16 | 0/16 | 0.31 | 0.29 | 0.23 | 0.17 | 0.21 | 0.28 | 0.25 |
| M2: E + L + g | TCH | 16 | 16/0 | 0.23 | 0.25 | 0.12 | 0.21 | 0.19 | 0.25 | 0.21 |
| M2: E + L + g | TCH | 16 | 12/4 | 0.2 | 0.23 | 0.18 | 0.24 | 0.2 | 0.26 | 0.22 |
| M2: E + L + g | TCH | 16 | 8/8 | 0.19 | 0.23 | 0.14 | 0.17 | 0.18 | 0.22 | 0.19 |
| M2: E + L + g | TCH | 16 | 4/12 | 0.13 | 0.14 | 0.07 | 0.11 | 0.09 | 0.15 | 0.12 |
| M2: E + L + g | TCH | 16 | 0/16 | 0.03 | 0.07 | 0.05 | 0.05 | 0.01 | 0.07 | 0.05 |
| M3: E + L + g + gE | SA | 16 | 16/0 | 0.61 | 0.51 | 0.43 | 0.35 | 0.38 | 0.48 | 0.46 |
| M3: E + L + g + gE | SA | 16 | 12/4 | 0.54 | 0.54 | 0.38 | 0.32 | 0.34 | 0.46 | 0.43 |
| M3: E + L + g + gE | SA | 16 | 8/8 | 0.53 | 0.48 | 0.35 | 0.28 | 0.22 | 0.43 | 0.38 |
| M3: E + L + g + gE | SA | 16 | 4/12 | 0.47 | 0.39 | 0.25 | 0.21 | 0.26 | 0.35 | 0.32 |
| M3: E + L + g + gE | SA | 16 | 0/16 | 0.32 | 0.25 | -0.05 | -0.03 | 0.12 | 0.17 | 0.13 |
| M3: E + L + g + gE | TCH | 16 | 16/0 | 0.21 | 0.27 | 0.13 | 0.21 | 0.21 | 0.25 | 0.21 |
| M3: E + L + g + gE | TCH | 16 | 12/4 | 0.18 | 0.26 | 0.18 | 0.23 | 0.2 | 0.26 | 0.22 |
| M3: E + L + g + gE | TCH | 16 | 8/8 | 0.18 | 0.25 | 0.14 | 0.16 | 0.21 | 0.21 | 0.19 |
| M3: E + L + g + gE | TCH | 16 | 4/12 | 0.09 | 0.19 | 0.05 | 0.08 | 0.14 | 0.16 | 0.12 |
| M3: E + L + g + gE | TCH | 16 | 0/16 | 0.05 | 0.12 | 0.03 | 0 | 0.08 | 0.07 | 0.06 |
| M1: E + L | SA | 12 | 12/0 | 0.47 | 0.4 | 0.37 | 0.37 | 0.31 | 0.44 | 0.39 |
| M1: E + L | SA | 12 | 8/4 | 0.26 | 0.23 | 0.22 | 0.21 | 0.23 | 0.26 | 0.23 |
| M1: E + L | SA | 12 | 4/8 | 0.2 | 0.18 | 0.16 | 0.17 | 0.13 | 0.2 | 0.17 |
| M1: E + L | SA | 12 | 0/12 | 0.03 | 0.02 | -0.01 | 0.01 | 0.03 | 0.05 | 0.02 |
| M1: E + L | TCH | 12 | 12/0 | 0.22 | 0.23 | 0.14 | 0.23 | 0.19 | 0.22 | 0.2 |
| M1: E + L | TCH | 12 | 8/4 | 0.13 | 0.17 | 0.15 | 0.16 | 0.18 | 0.23 | 0.17 |
| M1: E + L | TCH | 12 | 4/8 | 0.12 | 0.14 | 0.09 | 0.13 | 0.11 | 0.12 | 0.12 |
| M1: E + L | TCH | 12 | 0/12 | 0 | 0 | 0.01 | 0 | -0.02 | -0.02 | 0 |
| M2: E + L + g | SA | 12 | 12/0 | 0.63 | 0.56 | 0.48 | 0.44 | 0.38 | 0.58 | 0.51 |
| M2: E + L + g | SA | 12 | 8/4 | 0.36 | 0.34 | 0.29 | 0.25 | 0.27 | 0.34 | 0.31 |
| M2: E + L + g | SA | 12 | 4/8 | 0.34 | 0.31 | 0.25 | 0.24 | 0.2 | 0.31 | 0.27 |
| M2: E + L + g | SA | 12 | 0/12 | 0.29 | 0.25 | 0.21 | 0.14 | 0.12 | 0.2 | 0.2 |
| M2: E + L + g | TCH | 12 | 12/0 | 0.23 | 0.25 | 0.16 | 0.24 | 0.2 | 0.24 | 0.22 |
| M2: E + L + g | TCH | 12 | 8/4 | 0.15 | 0.19 | 0.16 | 0.18 | 0.19 | 0.25 | 0.19 |
| M2: E + L + g | TCH | 12 | 4/8 | 0.15 | 0.17 | 0.1 | 0.15 | 0.13 | 0.15 | 0.14 |
| M2: E + L + g | TCH | 12 | 0/12 | 0.02 | 0.08 | 0.03 | 0.02 | 0.03 | 0.06 | 0.04 |
| M3: E + L + g + gE | SA | 12 | 12/0 | 0.64 | 0.58 | 0.49 | 0.43 | 0.38 | 0.55 | 0.51 |
| M3: E + L + g + gE | SA | 12 | 8/4 | 0.39 | 0.37 | 0.29 | 0.2 | 0.26 | 0.32 | 0.31 |
| M3: E + L + g + gE | SA | 12 | 4/8 | 0.39 | 0.33 | 0.23 | 0.18 | 0.14 | 0.28 | 0.26 |
| M3: E + L + g + gE | SA | 12 | 0/12 | 0.34 | 0.31 | 0.03 | -0.04 | 0.11 | 0.12 | 0.14 |
| M3: E + L + g + gE | TCH | 12 | 12/0 | 0.22 | 0.29 | 0.16 | 0.25 | 0.24 | 0.26 | 0.24 |
| M3: E + L + g + gE | TCH | 12 | 8/4 | 0.15 | 0.22 | 0.16 | 0.18 | 0.21 | 0.26 | 0.2 |
| M3: E + L + g + gE | TCH | 12 | 4/8 | 0.14 | 0.2 | 0.09 | 0.13 | 0.16 | 0.14 | 0.14 |
| M3: E + L + g + gE | TCH | 12 | 0/12 | 0.06 | 0.16 | 0 | 0.01 | 0.09 | 0.05 | 0.06 |

Supplementary Table 2. Statistical significancy of model's performance differences. The following ANOVA test was run for i) all results and ii) only the results with the maximum calibration size (186 phenotypes in the train set): accuracy = model + environment + design. Tukey's HSD was then applied to the ANOVA object to test differences between models. No significant differences between models M2 and M3 were found except for TCH and maximum calibration size.

| **Dataset** | **trait** | **contrast** | **diff** | **lwr** | **upr** | **p.adj** |
| --- | --- | --- | --- | --- | --- | --- |
| all  calibration sizes | SA | M3-M2 | -0.001 | -0.023 | 0.02 | 0.988 |
|  |  | M1-M2 | -0.135 | -0.157 | -0.113 | 0 |
|  |  | M1-M3 | -0.134 | -0.155 | -0.112 | 0 |
|  | TCH | M3-M2 | 0.007 | -0.004 | 0.018 | 0.293 |
|  |  | M1-M2 | -0.023 | -0.034 | -0.011 | 0 |
|  |  | M1-M3 | -0.03 | -0.041 | -0.019 | 0 |
| max calibration  size (186) | SA | M3-M2 | -0.01 | -0.022 | 0.001 | 0.094 |
|  |  | M1-M2 | -0.14 | -0.152 | -0.129 | 0 |
|  |  | M1-M3 | -0.13 | -0.142 | -0.118 | 0 |
|  | TCH | M3-M2 | 0.007 | 0.002 | 0.013 | 0.008 |
|  |  | M1-M2 | -0.026 | -0.032 | -0.021 | 0 |
|  |  | M1-M3 | -0.033 | -0.039 | -0.028 | 0 |
